# Supplementary material for: Enhanced Symbiotic Characteristics in Bacterial Genomes with the Disruption of rRNA Operon
Source: Biology (Basel). 2020 Dec 3;9(12):440. doi: 10.3390/biology9120440 (PMC7761764; doi:10.3390/biology9120440)
Supplement: Supplementary file 1 [file biology-09-00440-s001.zip › Supplementary Table S2.pdf]

Supplementary Table S2. List of genera with the conservation of rRNA operon disruption.

Genus	Unlinked genomes*	Genome size (average, bp)	rRNA copy number		Characteristics	Habitat (host)	Reference
			16S	23S			
Anaplasma	**8**	**1349657.5**	**1**	**1**	Pathogen (humans, mammals; anaplasmosis)	Ticks	[1-3]
Borrelia	20	1192776.1	1	1	Pathogen (humans, mammals; relapsing fever)	Ticks, lice	[4-6]
Borreliella	16	1265183.6	1	1~2	Pathogen (humans; lyme disease)	Ticks, lice	[4,5,7-9]
Brachyspira	9	2976921.6	1	1	Pathogen (humans, mammals)	Various animals	[10-14]
Buchnera	24	592061.1	1	1	Symbiont (aphids)	Aphids	[15]
Dehalococcoides	25	1403003.4	1	1	Organohalide respiration	Environmental sediments	[16]
Ehrlichia	15	1260075.1	1	1	Pathogen (humans, mammals; ehrlichiosis)	Ticks	[17-20]
Eperythrozoon A	6	687613.3	1	1	Pathogen (mammals)	Arthropods	[21]
Gimesia	6	7922255.7	2	1	Isolated from ocean (not characterized)	Ocean	[22]
Helicobacter	175	1638038.3	1~2	1~2	Pathogen (humans, mammals)	Humans, mammals	[23-25]
Leptospira	29	4452983.9	2	2	Pathogen (humans, mammals; leptospirosis)	Stagnant water, mammals	[26]
Orientia	8	2171624.8	1	1	Pathogen (humans; typhus, Tsutsugamushi disease)	Mites	[27,28]
Rickettsia	68	1270779.6	1	1	Pathogen (humans; typhus)	Arthropods, nematodes	[28]
Thermus	15	2253012.2	2	2	High temperature tolerance	Hot water	[29]
TM7x	4	770656.0	1	1	Pathogen (bacteria)	Bacteria	[30]
Wolbachia	23	1308337.4	1	1	Symbiont (insects)	Nematodes	[31,32]

* Genera with only unlinked rRNA operons and with four or more genomes were included.

References

1. Munderloh, U.G.; Blouin, E.F.; Kocan, K.M.; Ge, N.L.; Edwards, W.L.; Kurtti, T.J. Establishment of the tick (Acari: Ixodidae)-borne cattle pathogen Anaplasma marginale (Rickettsiales: Anaplasmataceae) in tick cell culture. *Journal of medical entomology* **1996**, *33*, 656–664.
2. Stuen, S. Anaplasma phagocytophilum-the most widespread tick-borne infection in animals in Europe. *Veterinary research communications* **2007**, *31*, 79–84.
3. Ohashi, N.; Inayoshi, M.; Kitamura, K.; Kawamori, F.; Kawaguchi, D.; Nishimura, Y.; Naitou, H.; Hiroi, M.; Masuzawa, T. Anaplasma phagocytophilum–infected ticks, Japan. *Emerging infectious diseases* **2005**, *11*, 1780.
4. Barbour, A.G.; Hayes, S.F. Biology of Borrelia species. *Microbiological reviews* **1986**, *50*, 381.
5. Adeolu, M.; Gupta, R.S. A phylogenomic and molecular marker based proposal for the division of the genus Borrelia into two genera: The emended genus Borrelia containing only the members of the relapsing fever Borrelia, and the genus Borreliella gen. nov. containing the members of the Lyme disease Borrelia (Borrelia burgdorferi sensu lato complex). *Antonie Van Leeuwenhoek* **2014**, *105*, 1049–1072.
6. Krause, P.J.; Narasimhan, S.; Wormser, G.P.; Rollend, L.; Fikrig, E.; Lepore, T.; Barbour, A.; Fish, D. Human Borrelia miyamotoi infection in the United States. *The New England journal of medicine* **2013**, *368*, 291.
7. Baranton, G.; Postic, D.; Saint Girons, I.; Boerlin, P.; Piffaretti, J.-C.; Assous, M.; Grimont, P.A. Delineation of Borrelia burgdorferi sensu stricto, Borrelia garinii sp. nov., and group VS461 associated with Lyme borreliosis. *International Journal of Systematic and Evolutionary Microbiology* **1992**, *42*, 378–383.
8. Pritt, B.S.; Respicio-Kingry, L.B.; Sloan, L.M.; Schriefer, M.E.; Replogle, A.J.; Bjork, J.; Liu, G.; Kingry, L.C.; Mead, P.S.; Neitzel, D.F. Borrelia mayonii sp. nov., a member of the Borrelia burgdorferi sensu lato complex, detected in patients and ticks in the upper midwestern United States. *International journal of systematic and evolutionary microbiology* **2016**, *66*, 4878.
9. Schutzer, S.E.; Fraser-Liggett, C.M.; Qiu, W.-G.; Kraiczy, P.; Mongodin, E.F.; Dunn, J.J.; Luft, B.J.; Casjens, S.R. Whole-genome sequences of Borrelia bissettii, Borrelia valaisiana, and Borrelia spielmanii. Am Soc Microbiol: 2012.
10. Bellgard, M.I.; Wanchanthuek, P.; La, T.; Ryan, K.; Moolhuijzen, P.; Albertyn, Z.; Shaban, B.; Motro, Y.; Dunn, D.S.; Schibeci, D. Genome sequence of the pathogenic intestinal spirochete Brachyspira hyodysenteriae reveals adaptations to its lifestyle in the porcine large intestine. *PLoS ONE* **2009**, *4*.
11. Martínez-Lobo, F.J.; Hidalgo, Á.; García, M.; Argüello, H.; Naharro, G.; Carvajal, A.; Rubio, P. First identification of "Brachyspira hampsonii" in wild European waterfowl. *PLoS ONE* **2013**, *8*.
12. Pati, A.; Sikorski, J.; Gronow, S.; Munk, C.; Lapidus, A.; Copeland, A.; Del Tio, T.G.; Nolan, M.; Lucas, S.; Chen, F. Complete genome sequence of Brachyspira murdochii type strain (56-150 T). *Standards in genomic sciences* **2010**, *2*, 260–269.
13. Hampson, D.J. The spirochete Brachyspira pilosicoli, enteric pathogen of animals and humans. *Clinical microbiology reviews* **2018**, *31*, e00087–e00017.
14. Håfström, T.; Jansson, D.S.; Segerman, B. Complete genome sequence of Brachyspira intermedia reveals unique genomic features in Brachyspira species and phage-mediated horizontal gene transfer. *BMC genomics* **2011**, *12*, 395.
15. Moran, N.A.; Mira, A. The process of genome shrinkage in the obligate symbiont Buchnera aphidicola. *Genome biology* **2001**, *2*, research0054. 0051.
16. Löffler, F.E.; Yan, J.; Ritalahti, K.M.; Adrian, L.; Edwards, E.A.; Konstantinidis, K.T.; Müller, J.A.; Fullerton, H.; Zinder, S.H.; Spormann, A.M. Dehalococcoides mccartyi gen. nov., sp. nov., obligately organohalide-respiring anaerobic bacteria relevant to halogen cycling and bioremediation, belong to a novel bacterial class, Dehalococcoidia classis nov., order Dehalococcoidales ord. nov. and family Dehalococcoidaceae fam. nov., within the phylum Chloroflexi. *International journal of systematic and evolutionary microbiology* **2013**, *63*, 625–635.
17. Anderson, B.; Dawson, J.; Jones, D.; Wilson, K. Ehrlichia chaffeensis, a new species associated with human ehrlichiosis. *Journal of clinical microbiology* **1991**, *29*, 2838–2842.

18. Aguiar, D.M.; Cavalcante, G.T.; Pinter, A.; Gennari, S.M.; Camargo, L.M.A.; Labruna, M.B. Prevalence of Ehrlichia canis (Rickettsiales: Anaplasmataceae) in dogs and Rhipicephalus sanguineus (Acari: Ixodidae) ticks from Brazil. *Journal of medical entomology* **2007**, *44*, 126–132.
19. Frutos, R.; Viari, A.; Ferraz, C.; Morgat, A.; Eychenié, S.; Kandassamy, Y.; Chantal, I.; Bensaid, A.; Coissac, E.; Vachiery, N. Comparative genomic analysis of three strains of Ehrlichia ruminantium reveals an active process of genome size plasticity. *Journal of bacteriology* **2006**, *188*, 2533–2542.
20. Kawahara, M.; Ito, T.; Suto, C.; Shibata, S.; Rikihisa, Y.; Hata, K.; Hirai, K. Comparison of Ehrlichia muris strains isolated from wild mice and ticks and serologic survey of humans and animals with E. muris as antigen. *Journal of clinical microbiology* **1999**, *37*, 1123–1129.
21. Neimark, H.; Hoff, B.; Ganter, M. Mycoplasma ovis comb. nov.(formerly Eperythrozoon ovis), an epierythrocytic agent of haemolytic anaemia in sheep and goats. *International journal of systematic and evolutionary microbiology* **2004**, *54*, 365–371.
22. Scheuner, C.; Tindall, B.J.; Lu, M.; Nolan, M.; Lapidus, A.; Cheng, J.-F.; Goodwin, L.; Pitluck, S.; Huntemann, M.; Liolios, K. Complete genome sequence of Planctomyces brasiliensis type strain (DSM 5305 T), phylogenomic analysis and reclassification of Planctomycetes including the descriptions of Gimesia gen. nov., Planctopirus gen. nov. and Rubinisphaera gen. nov. and emended descriptions of the order Planctomycetales and the family Planctomycetaceae. *Standards in genomic sciences* **2014**, *9*, 10.
23. Suerbaum, S.; Michetti, P. Helicobacter pylori infection. *New England Journal of Medicine* **2002**, *347*, 1175–1186.
24. Harper, C.G.; Whary, M.T.; Feng, Y.; Rhinehart, H.L.; Wells, R.S.; Xu, S.; Taylor, N.S.; Fox, J.G. Comparison of diagnostic techniques for Helicobacter cetorum infection in wild Atlantic bottlenose dolphins (Tursiops truncatus). *Journal of clinical microbiology* **2003**, *41*, 2842–2848.
25. Dailidiene, D.; Dailide, G.; Ogura, K.; Zhang, M.; Mukhopadhyay, A.K.; Eaton, K.A.; Cattoli, G.; Kusters, J.G.; Berg, D.E. Helicobacter acinonychis: Genetic and rodent infection studies of a Helicobacter pylori-like gastric pathogen of cheetahs and other big cats. *Journal of bacteriology* **2004**, *186*, 356–365.
26. Adler, B.; de la Peña Moctezuma, A. Leptospira and leptospirosis. *Veterinary microbiology* **2010**, *140*, 287–296.
27. Seong, S.-Y.; Choi, M.-S.; Kim, I.-S. Orientia tsutsugamushi infection: Overview and immune responses. *Microbes and Infection* **2001**, *3*, 11–21.
28. Georgiades, K.; Merhej, V.; El Karkouri, K.; Raoult, D.; Pontarotti, P. Gene gain and loss events in Rickettsia and Orientia species. *Biology direct* **2011**, *6*, 6.
29. Omelchenko, M.V.; Wolf, Y.I.; Gaidamakova, E.K.; Matrosova, V.Y.; Vasilenko, A.; Zhai, M.; Daly, M.J.; Koonin, E.V.; Makarova, K.S. Comparative genomics of Thermus thermophilus and Deinococcus radiodurans: Divergent routes of adaptation to thermophily and radiation resistance. *BMC evolutionary biology* **2005**, *5*, 57.
30. Bor, B.; McLean, J.S.; Foster, K.R.; Cen, L.; To, T.T.; Serrato-Guillen, A.; Dewhirst, F.E.; Shi, W.; He, X. Rapid evolution of decreased host susceptibility drives a stable relationship between ultrasmall parasite TM7x and its bacterial host. *Proceedings of the National Academy of Sciences* **2018**, *115*, 12277–12282.
31. Parvizi, P.; Benlarbi, M.; Ready, P. Mitochondrial and Wolbachia markers for the sandfly Phlebotomus papatasi: Little population differentiation between peridomestic sites and gerbil burrows in Isfahan province, Iran. *Medical and veterinary entomology* **2003**, *17*, 351–362.
32. Hedges, L.M.; Brownlie, J.C.; O'Neill, S.L.; Johnson, K.N. Wolbachia and virus protection in insects. *Science* **2008**, *322*, 702–702.

.
