# Supplementary figures and images for: Enhanced Symbiotic Characteristics in Bacterial Genomes with the Disruption of rRNA Operon
Source: Biology (Basel). 2020 Dec 3;9(12):440. doi: 10.3390/biology9120440 (PMC7761764; doi:10.3390/biology9120440)

**A**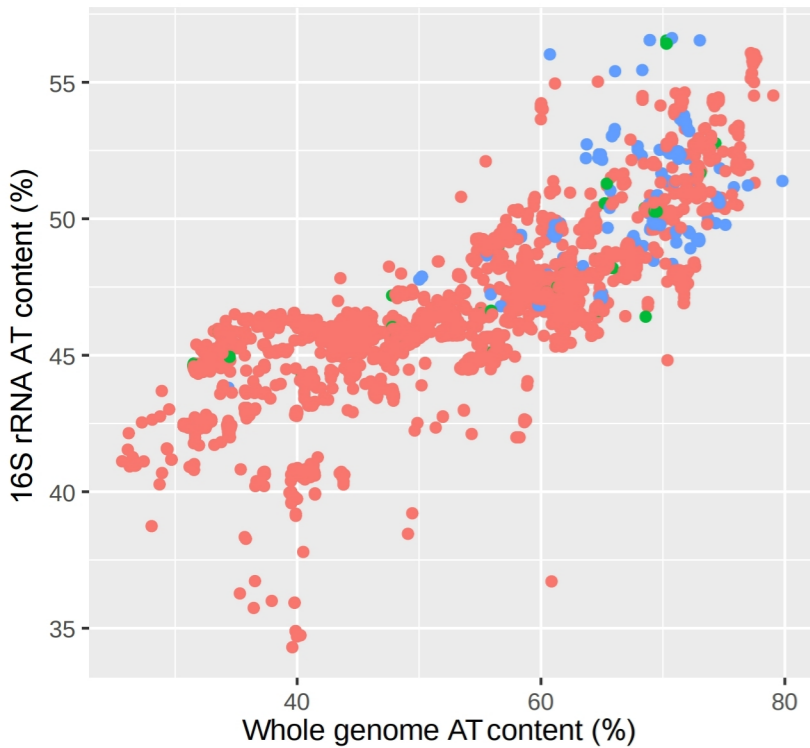**B**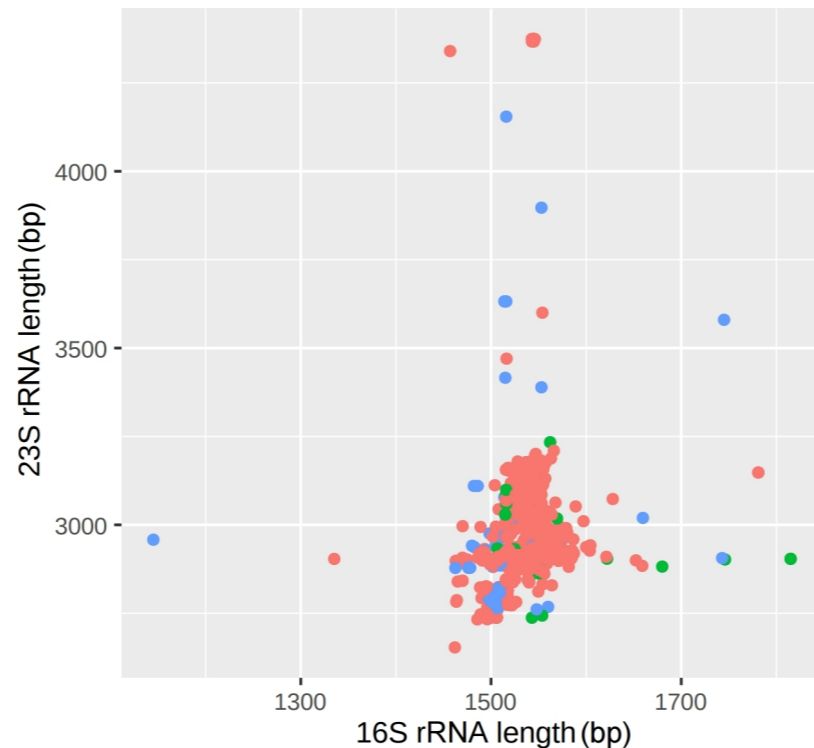**C**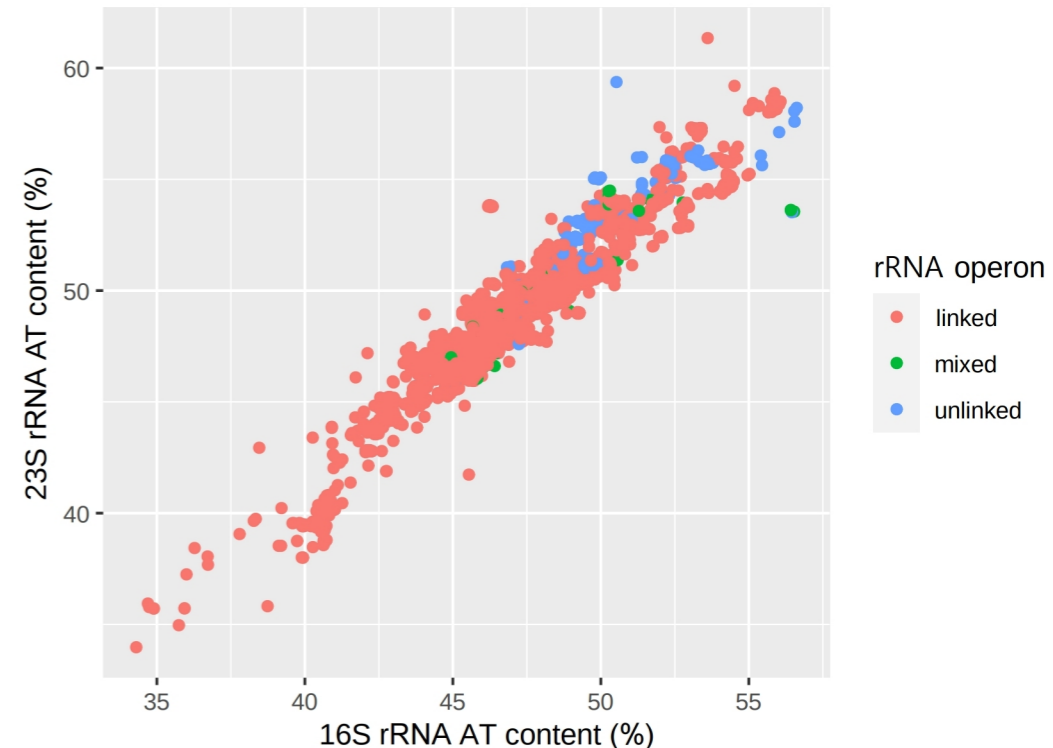

Supplement: Supplementary file 1 [file biology-09-00440-s001.zip › Supplementary Figure S1.pdf]

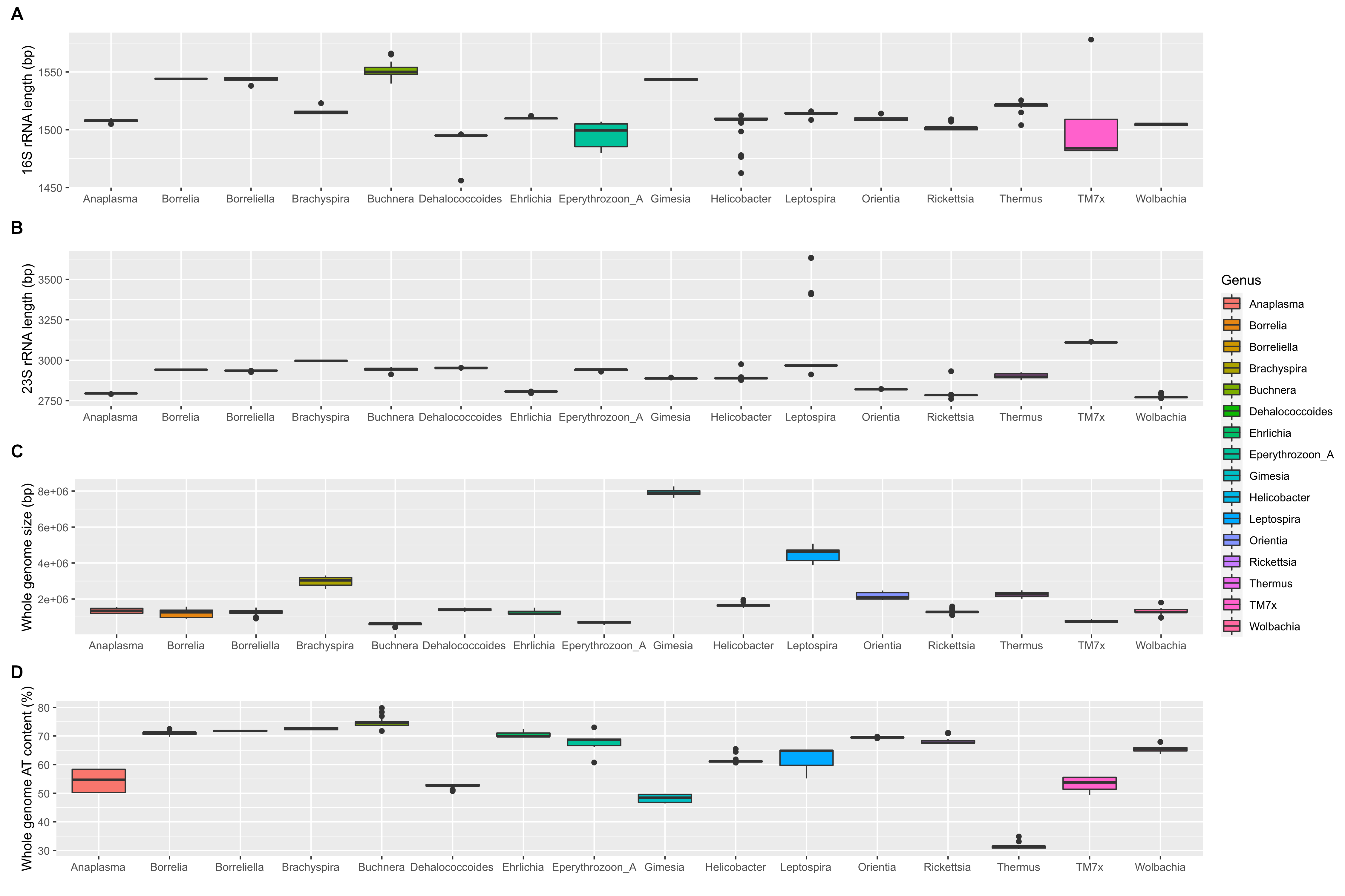

Supplement: Supplementary file 1 [file biology-09-00440-s001.zip › Supplementary Figure S2.png]
